# Adjuvant Proton Beam Radiation Therapy for Sinonasal Mucosal Melanoma

**DOI:** 10.1002/cnr2.70111

**Published:** 2025-02-05

**Authors:** Jamie S. K. Takayesu, Upendra Parvathaneni, George E. Laramore, Neil Panjwani, Jennifer Sillings, Neal D. Futran, Ian M. Humphreys, Aria Jafari, Waleed M. Abuzeid, Brittany Barber, Emily Marchiano, Sylvia M. Lee, John A. Thompson, Evan Hall, Shailender Bhatia, Cristina P. Rodriguez, Jay J. Liao

**Affiliations:** ^1^ Department of Radiation Oncology University of Washington Seattle Washington USA; ^2^ Department of Radiation Oncology University of Texas Medical Branch Galveston Texas USA; ^3^ Department of Otolaryngology‐Head and Neck Surgery University of Washington Seattle Washington USA; ^4^ Department of Hematology and Oncology University of Washington Seattle Washington USA

**Keywords:** immunotherapy, mucosal melanoma, protons, radiation therapy, sinonasal cancer

## Abstract

**Background:**

Head and neck mucosal melanoma (HNMM) is rare and carries a poor prognosis with high rates of disease progression. There is little data regarding the use of adjuvant proton radiation therapy in the management of sinonasal HNMM.

**Aims:**

We performed a retrospective review of patients with nonmetastatic sinonasal HNMM treated with adjuvant proton radiation from 2012 to 2022 at a single academic institution. Kaplan–Meier estimates were used for survival analyses.

**Methods and Results:**

Eight patients with sinonasal HNMM were treated with surgery and adjuvant proton radiation, and six received systemic therapy. Median follow‐up was 15 months (range: 3–68 months). Only one local failure was observed, and two patients developed distant metastases. Kaplan–Meier 1‐year results were as follows: local control 88%, distant metastasis‐free survival 75%, and overall survival 88%. No Grade 3 or higher late toxicities were observed.

**Conclusion:**

Surgical resection and adjuvant proton radiation provided early favorable local control and toxicity profiles in our cohort of patients with sinonasal HNMM. Further multi‐institutional work is needed to study this rare malignancy.

## 1 Introduction

Mucosal melanoma is a rare entity with an incidence of 2.20 per 1 000 000 person‐years in the United States. Mucosal melanoma is associated with a high risk of metastases and mortality [1]. Head and neck mucosal melanoma (HNMM) comprises about 40% of all mucosal melanoma cases [2, 3], with the sinonasal subsite being the most common [4]. Sinonasal HNMM carries a poor prognosis, with a 5‐year overall survival (OS) of about 25% [5, 6, 7]. A modern treatment paradigm for localized sinonasal HNMM that has been adopted at a number of centers includes primary endoscopic resection, adjuvant radiation, and consideration of adjuvant immunotherapy [8]. This approach has emerged largely from expert opinion and data from cutaneous melanoma.

There are mixed data regarding adjuvant radiation therapy for sinonasal HNMM [9, 10, 11, 12, 13]. The risk of local recurrence after surgery is high due to inconspicuous submucosal spread and difficulty achieving clear surgical margins. Adjuvant radiation is often recommended given the significant morbidity associated with local recurrence, which is often not salvageable. Even with adjuvant radiation, local control (LC) is only 50% at 5 years [9, 13].

There is limited data published on the outcomes of modern radiotherapy approaches in the multimodality treatment of sinonasal HNMM. Proton beam therapy provides a dosimetric advantage over x‐ray‐based approaches due to the lack of exit dose. In the sinonasal region, given the proximity of tumor and postoperative target volumes to numerous critical structures, proton therapy may improve coverage of high‐risk target volumes, while maximizing sparing of critical structures. This may lead to improved LC and reduce the risk of toxicities. A systematic review and meta‐analysis comparing the outcomes of patients with sinonasal cancer, including HNMM, treated with charged particle therapy (e.g., protons or carbon ion) vs. standard x‐ray therapy reported that 5‐year OS and disease‐free survival were higher for charged particle therapy, and that 5‐year disease‐free survival and locoregional control were higher for proton therapy vs. x‐ray‐based intensity‐modulated radiation therapy [14].

Advances in endoscopic surgical approaches decrease the morbidity of sinonasal surgery and increase complete surgical resection [15]. Advances in systemic therapy may have a role in reducing systemic progression and cytoreducing the primary. In patients with locoregionally advanced melanoma, randomized trials in resected node‐positive disease have demonstrated that adjuvant immunotherapy improves recurrence‐free survival and distant metastasis‐free survival (DMFS), although many of these trials excluded or had few patients with mucosal melanoma [16, 17, 18]. The use of immunotherapy for resected localized sinonasal HNMM has been extrapolated from these experiences, but the benefit is not clear.

Clinical data are limited regarding outcomes with protons as a component of the multimodality treatment of sinonasal HNMM. The goal of this study is to describe our institution's experience with postoperative proton therapy with and without adjuvant systemic therapy for sinonasal HNMM.

## 2 Materials and Methods

Institutional review board approval was obtained for this retrospective review. We identified nine patients treated with proton therapy for nonmetastatic sinonasal HNMM between 2012 and 2022 at a single academic institution. Data were collected retrospectively via the electronic medical record and the radiation treatment planning system.

### 2.1 Radiation Therapy

After the initial consult, patients underwent a CT simulation with a non‐contrast and contrast helical CT scan (1.25–2.5 mm slices) and immobilization with a head, neck, and shoulder thermoplastic mask, BoS headframe (Qfix, Avondale, Pennsylvania), custom MoldCare cushion and custom oral stent. Image registration and fusion with diagnostic preoperative and postoperative CT, MRI, and FDG PET‐CT scans were performed.

Tumor target volumes and organs at risk were delineated. The gross target volume was defined as any gross disease after surgery. Clinical target volume included the gross target volume and the presurgical volume with a margin, respecting anatomic boundaries. In some patients, a second clinical target volume was created to encompass a larger elective field to cover additional lower subclinical risk anatomic regions (e.g., adjacent or contralateral mucosal volumes in the nasal cavity or paranasal sinuses) and was prescribed to a lower dose.

All patients were treated with adjuvant proton therapy. One patient who had gross residual disease at the base of skull received a mixed beam approach of neutrons and protons with the intent to improve the LC of this radioresistant tumor. The prescribed proton doses used a standard relative biological effectiveness value of 1.1. Proton treatment was delivered with either uniform scanning (XiO version 5.00.02, XiO RTP System, Missouri, USA) or intensity‐modulated proton therapy with pencil beam scanning (RayStation, version 6.0, RaySearch Laboratories, Stockholm, Sweden). Planning target volume margin was variably used. Quality assurance included additional plan robustness analysis, based on ±3% range uncertainty and ±3 mm circumferentially for geometric uncertainty.

Imaged guided radiation therapy was performed by acquiring daily orthogonal 2‐D kV images and registered with two digitally reconstructed radiographs rendered from the planning CT. The patient positioning and verification system (Verisuite, Medcom, Germany) calculated the setup errors and provided six degrees of freedom correction vectors with sub‐millimeter accuracy. Translational setup errors were sent to the treatment control system to correct the treatment setup with a residual tolerance of 2 mm between the planned and treated setup.

### 2.2 Toxicity

Toxicity was retrospectively assessed and graded using the Common Terminology Criteria for Adverse Events version 5.0 based on the worst toxicity experienced by the patient over the follow‐up time. Acute toxicity was defined as toxicity that occurred either during radiation, or within 3 months of the last day of radiation. Late toxicity was defined as occurring more than 3 months after the last day of radiation.

### 2.3 Statistical Analysis

Data were reported using frequency and percentage for categorical variables and median and range for continuous variables. Survival times were calculated from the date of surgery. Progression‐free survival (PFS), LC, DMFS, and OS were estimated using the Kaplan–Meier method. For all Kaplan–Meier analyses, patients were censored at the last reported follow‐up. All *p* values are two‐sided and were considered statistically significant if less than 0.05. All statistical analyses were performed using R version 4.2.2 (R Foundation for Statistical Computing, Vienna, Austria).

## 3 Results

The baseline characteristics of the eight patients included in this study are detailed in Table 1. Most patients were male (*n* = 5, 63%) and White (*n* = 7, 88%). Most patients presented with nasal congestion (*n* = 5, 63%) and/or epistaxis (*n* = 5, 63%). Four patients (50%) had pT4 tumors, and four (50%) had pT3 tumors. Of the patients with pT4a disease, three had involvement of the nasopharynx and sphenoid sinuses. One patient with pT4b disease additionally had base of skull, maxillary sinus, and orbital involvement. All patients were clinically node‐negative at the time of surgery, but one patient progressed and became clinically node‐positive at the time of CT simulation. The median follow‐up was 15 months (range 3–68 months).

**TABLE 1:** Baseline patient characteristics.

Characteristic	Median (min–max) or *n* (%)
Age at diagnosis	65 (37–82)
Sex	
Male	5 (63%)
Female	3 (38%)
Race	
White	7 (88%)
Asian	1 (12%)
Ethnicity	
Hispanic	1 (12%)
Non‐Hispanic	7 (88%)
Presenting symptom (patient can present with more than one symptom)	
Nasal congestion	5 (63%)
Epistaxis	5 (63%)
Dyssomnia	1 (12%)
Size of primary (cm)	3.3 (2.2–5.8)
Location of primary	
Nasal cavity	6 (75%)
Nasopharynx	1 (12%)
Ethmoid sinus	1 (12%)
T stage	
pT3	4 (50%)
pT4a	3 (38%)
pT4b	1 (12%)
N stage	
cN0	7 (88%)
cN1	1 (12%)
Margin status	
Negative	3 (38%)
Positive	3 (38%)
Unknown	2 (25%)

### 3.1 Surgery

The primary tumor was either resected by an endoscopic or combined approach. Four patients underwent an orbital resection, and three had a skull base resection. Gross total resection was achieved in three patients, negative/close margin in three patients, and positive margin in three patients. There were no immediate major postoperative complications within this cohort and no reported wound dehiscence.

### 3.2 Radiation

The median time from surgery to the first day of radiation was 46 days (range: 33–67 days). All patients completed the planned radiotherapy course with no unplanned treatment breaks.

**FIGURE 1:**
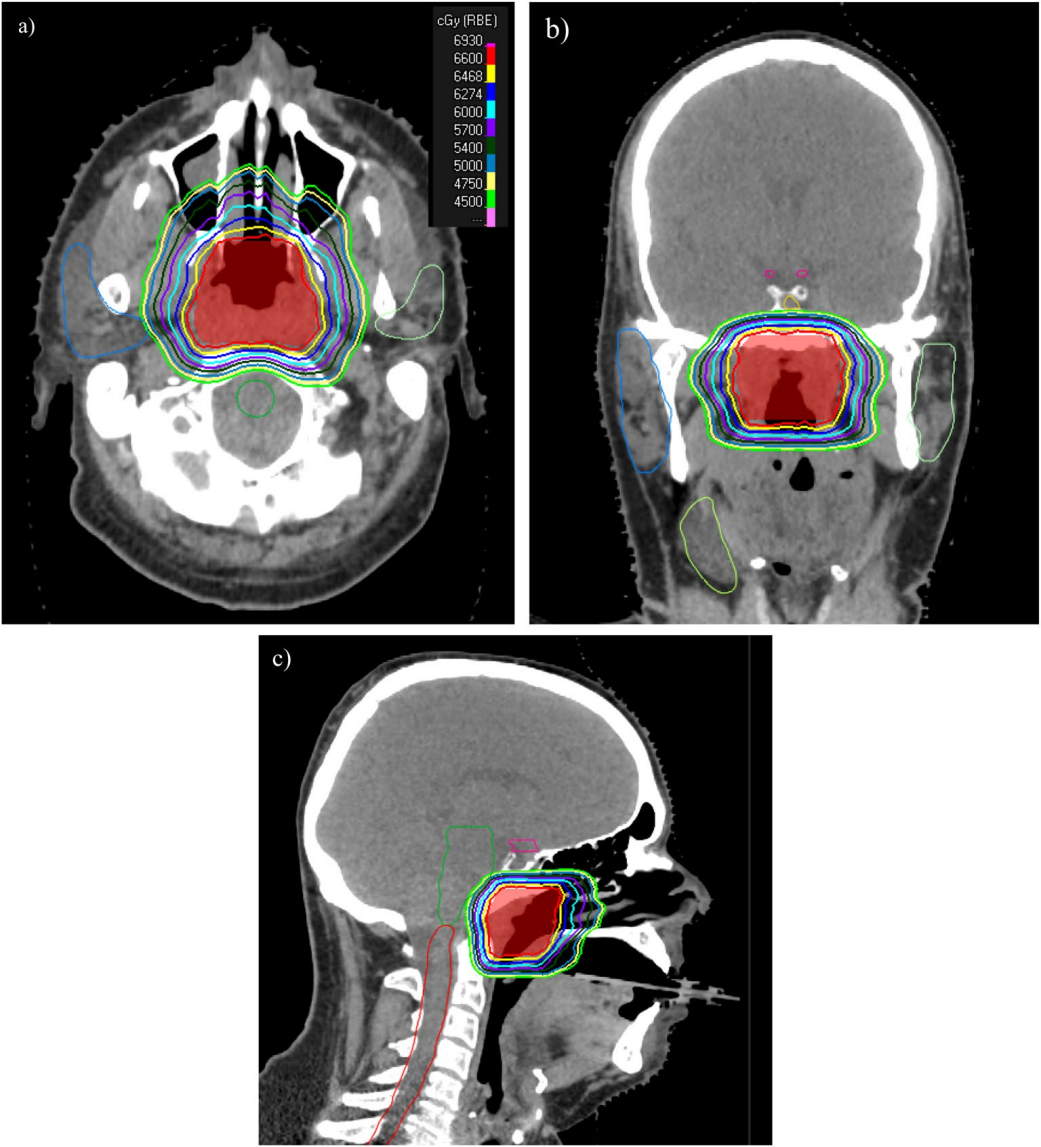
Representative radiation plan of a patient who presented with T3N0 sinonasal HNMM arising from and confined to the nasopharynx managed with an endoscopic resection with gross total resection but close surgical margins. The patient then received adjuvant proton radiation 66 GyE in 33 fractions to the nasopharynx. Planning target volume is shaded in red. Representative axial (a), coronal (b), and sagittal (c) slices are provided.

The most common fractionation was 60–66 GyE in 30–33 fractions given once daily. Two patients were treated with a moderately hypofractionated course of 48 GyE in 20 fractions, per treating physician's preference [19]. Lower‐risk subclinical volumes were typically dose‐painted to 54 GyE. Three cases also included ipsilateral cervical nodal coverage: one to ipsilateral levels Ib and retropharyngeal nodes given proximity to the primary after a pN0 elective neck dissection; one due to a radiographically suspicious new lymph node seen on the CT simulation that was not seen on initial staging scans; and the last per treating physician's preference. A representative proton plan is shown in Figure 1.

Overall, doses to the organs at risk were within the dose constraints recommended by the Qualitative Analysis of Normal Tissue Effects in the Clinic effort (Table 3). Only one patient had a temporal lobe maximum dose exceeding 60 GyE, due to the disease extent and location of the primary in the nasopharynx. Dose to the optic structures exceeded 54 GyE in one patient (59 GyE) due to the extent of disease in the orbit. In this same patient, the maximum dose to the right eye was also high at 67 GyE, but the mean dose to the right eye was 44 GyE. Parotid sparing was achieved in most patients, and oral cavity mean dose was generally less than 20 GyE. The median pituitary gland mean dose was 37 GyE (range: 16–49 GyE).

### 3.3 Radiation Toxicity

Treatment was well‐tolerated, with no Grade 4 or 5 adverse events (Table 4). Three patients developed Grade 3 dermatitis. Two patients developed Grade 3 mucositis, both of whom had elective nodal irradiation, including Level 1b. All skin and mucosal Grade 3 toxicities recovered normally after treatment. No patients required feeding tubes, and there were no emergency department visits or hospital admissions for acute radiation toxicity. Late toxicities are summarized in Table 4. There were no Grade 2 or 3 late toxicities, specifically no high‐grade visual or hearing complications and no brain necrosis or osteoradionecrosis. Sinus problems and eye tearing were the most common long‐term side effects of radiation, which were managed conservatively with lifestyle changes and medical management.

### 3.4 Systemic Therapy

Six patients (75%) received systemic therapy as part of definitive treatment (Table 2). The most common treatment was adjuvant PD‐1 inhibitor immunotherapy; three patients received nivolumab and one received pembrolizumab. One patient received cytotoxic neoadjuvant chemotherapy at an outside institution with vincristine, doxorubicin, cyclophosphamide, ifosfamide, and etoposide given an initial misdiagnosis of Ewing's sarcoma. Final surgical pathology during resection was then interpreted as HNMM. One patient with a BRAF V600E mutation received adjuvant dabrafenib/trametinib. Another patient received concurrent and adjuvant pembrolizumab per provider recommendation, because the patient presented with T4a disease and had gross residual disease postoperatively.

**TABLE 2:** Treatment characteristics.

	Median (range) or *n* (%)
Surgical management of the primary	
Endoscopic approach only	3 (38%)
Endoscopic and transorbital approach	2 (25%)
Other combined approach	3 (38%)
Surgical management of the neck	
None	0
Adjuvant radiation—Type	
Proton radiation	7 (88%)
Mixed beam proton/neutron radiation	1 (11%)
Adjuvant radiation—Duration (days)	42 (25–48)
Adjuvant radiation—Dose (GyE)	62 (36–66)
Adjuvant radiation—Fractions	30 (18–33)
Adjuvant radiation—Nodal irradiation	2 (25%)
Systemic therapy—Timing	
Neoadjuvant	1 (12%)
Concurrent/adjuvant	1 (12%)
Adjuvant	5 (63%)
Systemic therapy—Agent	
Multi‐agent chemotherapy	1 (12%)
Pembrolizumab	1 (12%)
Nivolumab	3 (38%)
Dabrafenib/trametinib	1 (12%)
Cycles of adjuvant immunotherapy received	4 (2–12)
Reasons for discontinuing adjuvant systemic therapy	
Disease progression	2 (25%)
Completed treatment	1 (12%)
Toxicity	1 (12%)
Ongoing treatment	1 (12%)

**TABLE 3:** Dosimetry data.

	Median (range)
CTV^a^ D95% (%)	1.01 (0.87–1.01)
CTV^a^ D98% (%)	0.99 (0.71–1.01)
PTV^b^ D95% (%)	1 (0.76–1.01)
Right optic nerve max dose (Gy)	36.63 (14.62–59.65)
Right optic nerve mean dose (Gy)	17.66 (8.4–51.92)
Left optic nerve max dose (Gy)	44.72 (21.31–51.84)
Left optic nerve mean dose (Gy)	31.37 (10.92–41.83)
Optic chiasm max dose (Gy)	35.21 (9.96–47.04)
Optic chiasm mean dose (Gy)	15.84 (2.7–29.56)
Oral cavity mean dose (Gy)	14.61 (9.48–21.07)
Right eye max dose (Gy)	43.33 (14.01–67.13)
Right eye mean dose (Gy)	15.55 (2.27–43.9)
Left eye max dose (Gy)	50.15 (14.09–52.44)
Left eye mean dose (Gy)	20.52 (1.91–30.79)
Right cochlea max dose (Gy)	5.99 (0.46–35.75)
Left cochlea max dose (Gy)	16.82 (3.67–32.29)
Right temporal lobe max dose (Gy)	49.62 (8.57–65.41)
Right temporal lobe V60Gy (cc)	0 (0–0.1)
Left temporal lobe max dose (Gy)	49.76 (13.22–63.01)
Left temporal lobe v60Gy (cc)	0 (0–0)
Pituitary mean dose (Gy)	37.06 (15.57–48.57)
Right parotid mean dose (Gy)	4.29 (0.02–48.05)
Left parotid mean dose (Gy)	26.11 (2–43.68)

^a^ Clinical target volume.

^b^ Planning target volume.

**TABLE 4:** Radiation toxicity Common Terminology Criteria for Adverse Events v.5.0.

	Grade 1	Grade 2	Grade 3
Acute toxicities			
Fatigue	6	0	0
Anorexia	1	0	0
Nausea	0	0	0
Dysgeusia	4	1	0
Oral mucositis	2	3	2
Oral pain	2	3	0
Dry mouth	4	1	0
Dysphagia	3	0	0
Esophageal pain	1	0	0
Headache	2	0	0
Tinnitus	0	0	0
Hearing impairment	1	0	0
Vision changes	0	0	0
Dry eye	6	0	0
Trismus	2	0	0
Dyssomnia	2	0	0
Sinus disorder	5	0	0
Nasal congestion	1	2	0
Rhinorrhea	6	0	0
Skin changes	4	2	2
Late toxicities			
Vision changes	0	0	0
Epiphora	4	0	0
Hearing	0	0	0
Sinus issues (decreased smell, post‐nasal drip, rhinorrhea or nasal congestion)	5	0	0
Epistaxis	5	0	0
Lymphedema	1	0	0
Brain necrosis	0	0	0

All five patients who received adjuvant systemic therapy were advised to stay on therapy for at least 12 months. One patient completed the 12 months of adjuvant therapy. One patient on adjuvant pembrolizumab and another on adjuvant nivolumab were taken off adjuvant immunotherapy due to disease progression after two and one cycle, respectively. One patient discontinued adjuvant nivolumab after two cycles due to Grade 2 colitis and myalgias, and another discontinued pembrolizumab after one cycle due to Grade 3 bullous pemphigoid.

### 3.5 Oncologic Outcomes

One local failure, no regional failures, and two distant failures occurred. One patient had persistent disease after adjuvant radiation and had in‐field progression 4 months after completing adjuvant radiation, while on adjuvant nivolumab. After 1 month, he developed distant metastases involving the lung, retroperitoneum, pelvis, and bones. The patient was switched to dual therapy with ipilimumab and nivolumab, but passed away 3 months after being diagnosed with metastatic disease. Another patient developed multiple liver metastases 2 months after surgery and adjuvant radiation, while on adjuvant pembrolizumab. The patient was switched to ipilimumab and nivolumab and has had no evidence of progression for the past 10 months. Six patients had no evidence of disease after completing radiation, of which four were started on adjuvant immunotherapy or targeted therapy, and one completed all planned 12 months of adjuvant therapy.

**FIGURE 2:**
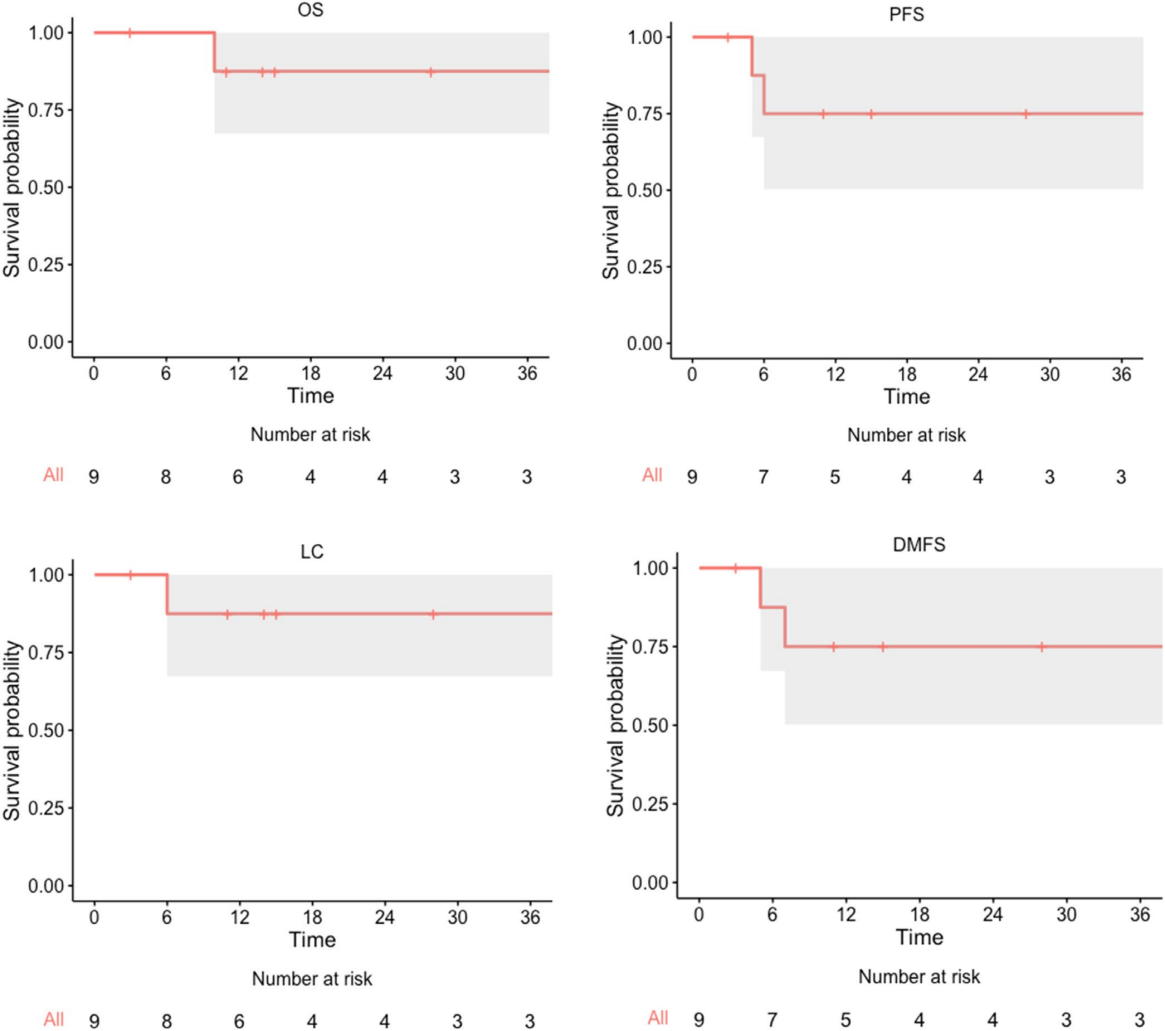
Kaplan–Meier survival curves for overall survival (OS), progression‐free survival (PFS), local control (LC), and distant metastasis‐free survival (DMFS).

Using the Kaplan–Meier method, 1‐year and 2‐year OS were both estimated to be 88% (95% CI: 67%–100%) (Figure 2). One‐year and 2‐year LC were both 88% (95% CI: 67%–100%). One‐year and 2‐year DMFS were both 75% (95% CI: 63%–100%).

One patient had 2 years of follow‐up without evidence of recurrence, and two additional patients had 5 years of follow‐up without evidence of recurrence. These three long‐term survivors all had pT3 disease. One of the long‐term survivors had a radiographically enlarged node found during radiation planning that was not resected and was boosted with radiation. Two of the three patients had microscopically positive margins. All were treated with conventionally fractionated radiation. One of the long‐term survivors completed 12 months of adjuvant nivolumab, whereas two did not receive adjuvant systemic therapy.

## 4 Discussion

Our study describes a cohort of patients with sinonasal HNMM managed with modern multimodality treatment with endoscopic resection and adjuvant proton radiation, with most receiving adjuvant systemic therapy. This approach yielded encouraging early outcomes with a 1‐year LC of 88% and OS of 88%. Radiation therapy was well tolerated with no Grade 3 or higher late toxicities.

Improvements to local therapy are needed. Single‐institution series report 2‐year LRFS of around 60% and 2‐year OS of 50%–60% [7, 20, 21, 22]. The historically suboptimal LC seen in HNMM may be related to the prevalence of locally advanced disease at presentation, difficulty achieving clear surgical margins, the proximity of numerous critical structures, and the relative radioresistance of melanoma. LC is particularly important with sinonasal HNMM since local recurrences may be associated with significant morbidity and may not be salvageable with further local therapy.

Multiple meta‐analyses in HNMM suggest that the addition of adjuvant radiation after surgical resection improves LRFS, although without a clear impact on OS [23, 24, 25], especially in desmoplastic and resected node‐positive melanoma [19, 26]. In HNMM, adjuvant radiation therapy may have a greater magnitude of benefit, as demonstrated in a prior meta‐analysis [25]. LC is particularly important in the head and neck, as a local recurrence can cause bleeding, pain, or disfigurement, all of which can significantly deteriorate patients' quality of life. Several centers have attempted to further improve LC with radiation with the use of high linear energy transfer particle beam radiation including carbon ion and neutron therapy [27, 28, 29].

Proton therapy has dosimetric advantages over x‐ray therapy due to the rapid dose fall‐off distal to the target that allows for better sparing of nearby organs at risk, which may reduce toxicity, while allowing for possible improved target coverage and potential dose escalation [30, 31]. A systematic review and meta‐analysis of paranasal sinus and nasal cavity malignancies reported that proton therapy compared to photons resulted in improved 5‐year OS, 5‐year disease‐free survival, and locoregional control at longest follow‐up [14]. Multiple single institution studies using charged particle therapy have reported high rates of LC, exceeding 80% at 2 years, and rates of Grade 3 or higher late toxicity in the range of 2%–22%, which compares favorably with photon‐based series [32, 33, 34, 35, 36, 37, 38]. Our findings show a favorable toxicity profile and low incidence of high‐grade acute and late toxicities for proton therapy in the postoperative setting.

There have been a few series looking at proton therapy for HNMM. Several studies from Japan describe high rates of LC between 75% and 95% at 1 year in unresectable HNMM treated definitively with proton or carbon ion radiation [29, 39, 40]. To our knowledge, there have only been two published experiences on proton therapy in the adjuvant setting for resectable HNMM. One study from the University of Florida included 21 patients, including five sinonasal cases, treated with definitive or adjuvant radiation, but no adjuvant immunotherapy was used [41]. The 5‐year LC was 79%, 5‐year DMFS was 20%, and 5‐year OS was 22%. Three patients had high‐grade radiation toxicities, including bilateral blindness and skin necrosis. A Japanese study described 62 patients with HNMM (*n* = 56 sinonasal) treated definitively or adjuvantly with proton or carbon ion therapy [29]. Two‐year LC was 78%, which is higher than historical results reported in x‐ray studies.

Adjuvant PD‐1 immunotherapy has been shown to significantly improve RFS in patients with high‐risk resected cutaneous malignant melanoma [16, 42, 43]. CheckMate 238 was the only large Phase III study in this space to include mucosal melanoma, and there were only 29 patients with this subtype [18]. Ipilimumab/nivolumab in the adjuvant setting has also been examined in a small series for mucosal melanoma, although in a single‐arm study design [44]. Metastatic mucosal melanoma has been observed to be resistant to systemic immunotherapy approaches compared to cutaneous melanoma [45, 46]. Given the small sample size of our study, it is difficult to draw firm conclusions regarding the efficacy of adjuvant immunotherapy for sinonasal HNMM. The two patients in our series who developed distant metastases progressed while on adjuvant immunotherapy. Further work is needed to evaluate systemic therapy agents, determine patient selection, and define the optimal timing of systemic therapy.

The limitations of our study include the small size and retrospective nature. However, this is one of the few series that describes the use of adjuvant proton therapy for sinonasal HNMM. We present a favorable patient population with resectable disease who were fit enough for surgery and received proton radiotherapy, which could contribute to the good outcomes reported herein. We did not include patients treated with photon‐based therapy, so there could be selection biases between protons vs. photon‐treated patients. However, the practice at our institution has been to discuss all patients with HNMM at a multidisciplinary tumor board, and to refer all patients with sinonasal cancers for consideration of adjuvant proton radiation. The primary reason patients received photon‐based rather than proton‐based therapy was because of insurance coverage, though this was uncommon.

Median follow‐up is relatively short, so encouraging early disease control may not reflect long‐term outcomes. However, because the median survival for sinonasal mucosal melanoma is only 18 months, 1‐year, and 2‐year outcomes are clinically meaningful [47]. The potential risk of marginal recurrence or miss has been raised with protons given the sharp dose gradients. However, reassuringly, just one local failure was observed, and no marginal or regional recurrences were seen. It is possible our routine of treating the involved sub‐site to lower dose levels and proton plan robustness testing are factors that mitigate the risk of marginal recurrence. Finally, from a healthcare economics perspective, the potential advantages of proton therapy need to be weighed against the economic costs to the patient as well as the healthcare system.

## 5 Conclusion

Multimodality therapy with endoscopic resection followed by adjuvant proton radiation therapy with or without adjuvant systemic therapy yielded encouragingly high rates of early LC in sinonasal HNMM. Three patients in our series reached more than 2 years of follow‐up and remain without evidence of disease. These data highlight the potential for using protons to maximize the therapeutic index in this disease, and clinicians should consider a multidisciplinary approach as described. Additional work is warranted to define the optimal systemic therapy regimen and timing in relationship to local therapy. Longer follow‐up is needed, and additional multi‐institutional work should be done to increase sample sizes to study this rare malignancy.

## Author Contributions

**Jamie S. K. Takayesu:** conceptualization, data curation, formal analysis, investigation, methodology, resources, visualization, writing – original draft, writing – review and editing. **Upendra Parvathaneni:** data curation, writing – review and editing. **George E. Laramore:** data curation, writing – review and editing. **Neil Panjwani:** data curation, writing – review and editing. **Jennifer Sillings:** data curation, writing – review and editing. **Neal D. Futran:** data curation, writing – review and editing. **Ian M. Humphreys:** data curation, writing – review and editing. **Aria Jafari:** data curation, writing – review and editing. **Waleed M. Abuzeid:** data curation, writing – review and editing. **Brittany Barber:** data curation, writing – review and editing. **Emily Marchiano:** data curation, writing – review and editing. **Sylvia M. Lee:** data curation, writing – review and editing. **John A. Thompson:** data curation, writing – review and editing. **Evan Hall:** data curation, writing – review and editing. **Shailender Bhatia:** data curation, writing – review and editing. **Cristina P. Rodriguez:** data curation, writing – review and editing. **Jay J. Liao:** conceptualization, data curation, formal analysis, methodology, resources, writing – original draft, writing – review and editing.

## Ethics Statement

Ethical approval for this study was obtained from the University of Washington Institutional Review Board.

## Consent

Informed consent for patient information to be published in this article was not obtained because of the retrospective nature of the study and minimal harm as determined by the University of Washington Institutional Review Board.

## Conflicts of Interest

The authors declare no conflicts of interest.

## Data Availability

The data that support the findings of this study are available from the corresponding author upon reasonable request.
